# Les synovites villonodulaires du genou: à propos de 20 cas

**DOI:** 10.11604/pamj.2017.28.86.9507

**Published:** 2017-09-27

**Authors:** Omar Margad, Jalal Boukhris, Ouahb Azriouil, Mohamed Daoudi, Aziz Mortaji, Khalid Koulali

**Affiliations:** 1 Service de Traumatologie Orthopédie de l’Hôpital Militaire Avicenne, Marrakech, Maroc

**Keywords:** Synovite villonodulaire pigmentée, genou, synovectomie, Pigmented villonodular synovitis, knee, synovectomy

## Abstract

La synovite villonodulaire pigmentée (SVNP) est une prolifération bénigne rare de la synoviale des articulations, des bourses séreuses et des gaines tendineuses, d'étiopathogénie inconnue. Notre travail porte sur 20 cas de SVN du genou colligés à l'hôpital militaire Avicenne de Marrakech sur une période de 9 ans allant de janvier 2000 au décembre 2009 Il vise à identifier les spécificités de cette lésion, et à étudier ses principaux aspects anatomocliniques et pronostiques. L'incidence annuelle était de 2,2 cas par an. Ils étaient 15 hommes et 5 femmes, d'âge moyen de 32,5 ans, atteints du coté droit dans 55%des cas sous un mode mono articulaire chez 18 patients et bi articulaire chez un seul. La douleur et la tuméfaction étaient présentes dans 80% des cas, une masse palpable dans un cas, un syndrome méniscal a été retenu dans un cas, une mono arthrite septique dans 3 circonstances de même qu'un kyste poplité dans 2 autres. L'atteinte était diffuse dans 14 cas (70%), localisée dans 6 cas. L'imagerie par résonnance magnétique(IRM) pratiquée chez 5 patients était évocatrice chez 3, l'arthroscopie diagnostique a été utilisée chez 2 malades. La confirmation s'est faite à chaque fois à l'examen anatomopathlogique. Le traitement a consisté en une synovectomie subtotale dans 15 cas et en l'exérèse de la tumeur dans les autres formes localisées, 2 cas présentant une destruction ostéocartilagineuse ont nécessité une arthroplastie. L'évolution a été marquée par la survenue de 2 récidives sous la forme diffuse avec un recul de 3, 7 ans. On a noté une raideur avec atrophie quadricipitale chez 3 patients et une arthrolyse a été réalisée. Un cas de SVN confirmé par l'histologie s'est révélé être 5 mois après la synovectomie totale un Synovialosarcome monophasique envahissant l'os d'où l'indication de l'amputation.

## Abstract (en)

Pigmented villonodular synovitis (PVNS) is a rare benign proliferation of synovial joints, serous bursa, tendinous sheaths of unknown etiopathogeny. We here report 20 cases of PVNS of the knee recorded at the Avicenne Military Hospital, Marrakech over a period of 9 years, from January 2000 to December 2009. This study aimed to identify the specific features of this lesion and to examine its anatomoclinic and prognostic aspects. Annual incidence was 2.2 cases per year: 15 men and 5 women. The average age was 32.5 years. It occurred in the right-hand in 55%, 18 patients had monoarticular presentation of the disease while 1 patient had biarticular presentation of the disease. 80% of cases had pain and swelling, palpable mass was detected in 1 case, meniscal syndrome in 1 case, monoseptic arthritis in 3 cases while popliteal cyst in 2 cases. 14 cases (70%) had diffuse involvement, 6 cases had localized involvement. MRI was evocative in 3 patients out of 5; 2 patients underwent diagnostic arthroscopy. Diagnosis was based on anatomo-pathological examination. Treatment was based on subtotal synovectomy in 15 cases and on tumor excision in patients with localized involvement. 2 cases with osteocartilaginous destruction underwent arthroplasty. Patients’ evolution was marked by 2 diffuse recurrences after a mean follow-up of 3-7 years. 3 patients had stiffness associated with quadriceps atrophy, therefore arthrolysis was performed. One case of histologically confirmed PVNS had proved to be a monophasic synovial sarcoma invading the bone 5 months after total synovectomy. Hence, the indication for amputation.

## Introduction

La SVN est une tumeur bénigne de la synoviale à fort pouvoir prolifératif et destructeur pour les structures avoisinantes, à potentiel de récidive locale particulièrement élevé et dont l'origine semble histiocytaire ou fibroblastique [1,2]. Elle est caractérisée par une symptomatologie souvent non spécifique, l'examen par l'IRM permet désormais une bonne approche diagnostique, et la confirmation se fait à l'examen anatomopathologique. Néanmoins, elle pose le problème de diagnostic différentiel avec les autres lésions particulièrement le Synovialosarcome tant sur le plan clinique, radiologique, qu'histologique [3]. Cette étude se propose d'identifier les spécificités de cette lésion parmi les tumeurs et les dystrophies de la synoviale, et de contribuer à une mise au point sur les principaux aspects anatomocliniques et pronostiques de cette affection encore mystérieuse

## Méthodes

Il s'agit d'une étude rétrospective réalisée dans le service de traumato-orthopédie de l'hôpital Militaire Avicenne de Marrakech portant sur une série de 20 cas de SVN du genou sur une période de 9 ans allant de Janvier 2000 à Décembre 2009. Ce travail s'est basé sur les données des dossiers médicaux des patients et l'analyse d'une fiche d'exploitation (données anamnestiques, cliniques, paracliniques, thérapeutiques et évolutives).

## Résultats

**Figure 1:**
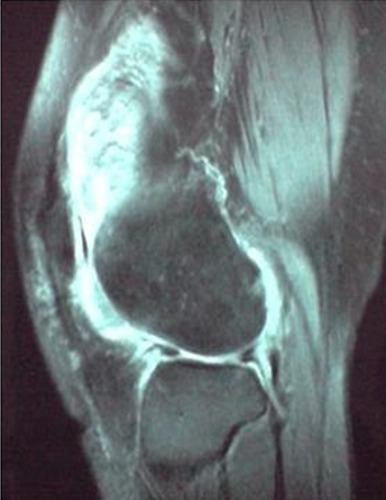
Aspect IRM de SVN diffuse

**Figure 2:**
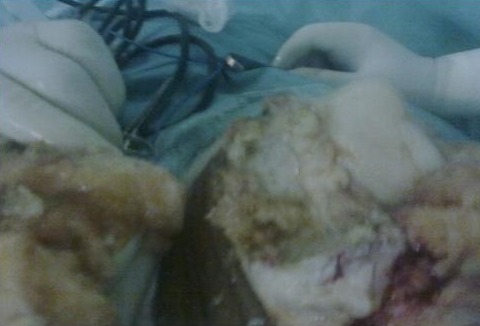
Aspect per opératoire de SVN

L'incidence retrouvée était de 2,2 cas par an, l'âge moyen était de 32,5 ans avec des extrêmes allant de 20 à 60 ans, Les patients se répartissaient en15 hommes (75%) et 5 femmes (15%). Le coté droit était atteint dans11 cas (55%) contre 9 cas pour le coté gauche, l'atteinte était mono articulaire chez 18 malades soit 90% des cas, bilatérale dans un cas .une notion de traumatisme du genou atteint a été relevée chez 3 patients, Dans notre série, le délai moyen avant la consultation était de 15 mois avec un intervalle entre 1mois et 5 ans, le tableau clinique était varié : la douleur et la tuméfaction étaient présentes dans 80% des cas, d'installation chronique, une douleur brutale a été notée chez 3 malades. Le blocage a été noté dans 5 cas faisant suspecter une lésion méniscale dans un cas et un corps étranger intra articulaire dans un autre. A l'examen clinique, l'épanchement articulaire a été présent dans 65% des cas, la limitation de la mobilité dans 6 cas, une amyotrophie quadricipitale dans 3 cas, une déformation en genu varum dans un cas. Une masse tumorale palpable existait dans un cas, de même qu'un kyste poplité dans 2 cas, et une mono arthrite dans 3 cas. La radiologie standard réalisée dans 16 cas était normale dans 9 cas, alors qu'elle objectivait des lésions géodiques dans un cas, un pincement articulaire dans 2 cas une déminéralisation osseuse diffuse dans un cas, une pseudo fracture du plateau tibial a été vu chez une patiente, ainsi qu'une arthrose avec destruction du compartiment fémorotibial externe chez une autre. La tomodensitométrie(TDM) faite chez 5 patients avait montré une hypertrophie synoviale chez 2 patients, quant à l'IRM (Figure 1), sollicitée dans 5 fois, elle a été évocatrice à chaque fois montrant une chondromatose dans un cas et une dégénérescence articulaire avec luxation du ménisque interne dans un autre. L'atteinte était diffuse dans 14 cas soit 70%, contre 6 formes localisée (30%). 2 malades ont bénéficié de l'arthroscopie diagnostique qui a évoqué macroscopiquement la maladie et a permis la biopsie. Sur le plan histologique, les résultats anatomopathologiques étaient identiques dans les formes diffuses et localisées, objectivant dans la plupart des cas, une hyperplasie des franges synoviales sur un chorion fibreux associée à des infiltrats inflammatoires à prédominance mononuclées avec ou sans pigments héminiques au sein des sidérophages, les cellules géantes étant inconstantes. La synovectomie totale (Figure 2) était le traitement envisagé chez 15 patients, la biopsie exérèse était faite pour les autres formes localisées, les 2 cas de destruction osseuses ont nécessité une arthroplastie totale. On a déploré 2 récidives compliquant 2 formes diffuses, et identifiées cliniquement par la douleur, et l'hydarthrose à répétition dans l'une, l'autre était une forme pseudo septique évoquant une mono arthrite septique. La radiographie avait montré dans les 2 cas un pincement articulaire. Ces récidives étaient des SVN diffuses dont le traitement procédé était une synovectomie subtotale. Par ailleurs une raideur a été notée dans 3 cas associée à une atrophie quadricipitale de 3cm chacune et une arthrolyse a été nécessaire permettant une amélioration de la mobilité articulaire.

## Discussion

L'incidence annuelle de la SVN est estimée à 1,8 cas par million d'habitants [4]. Elle touche les 2 sexes de façon égale [2, 4,5], notre série montre une prédominance masculine (80%) avec un sexe ratio de 3, Myers [4], LeTiec [6] et Sharma [7] retrouvent la même tendance mais l'inverse a été rapporté par d'autres auteurs [6]. L'âge moyen dans notre série est de 32,5 ans, pour 36 ans dans les cas publiés [6]. L'atteinte est quasi mono articulaire, exceptionnellement bi articulaire symétrique, notre étude trouve une atteinte du genou bilatérale dans 1 cas, seulement 11cas ont été dénombrés dans la littérature par Flandry [5]. La forme localisée représente 30% dans notre série, entre 20 et 25% dans les séries de Myers [4] et Olgivie-Harris[8], 33,3% dans la série de Gaubert [9], par contre la série de J.Dines et al (2007) [10] montre que la forme localisée est 7 fois plus fréquente que celle diffuse. La symptomatologie de la SVN est rarement caractéristique, le délai avant la consultation était variable en moyenne 15, 2 mois (1 mois-5ans), il peut aller jusqu'à 72 ans pour Rao [2], Myers [4] retrouve un délai plus cours (10-15mois). La forme diffuse se manifeste classiquement par un épanchement récidivant avec des douleurs le plus souvent modérées et chroniques ce qui est largement rencontré dans la majorité des séries étudiées [1, 7, 9,10]. Ainsi que dans notre étude, Howie et al [11] ont rapporté 3 cas de manifestations inaugurales aigue, traduisant une torsion avec infarcissement des nodules et c'était le cas chez 3 de nos malades. Quant à la forme localisée ; la symptomatologie est principalement mécanique, donnant le change avec un syndrome méniscale ou un corps étranger intra articulaire. Un syndrome méniscal a été retenu dans 1 cas dans notre étude, l'arthroscopie a découvert une lésion méniscale franche et une synovite villonodulaire localisée associée, cette association est exceptionnellement rapportée dans la littérature, elle a été vue dans 2 cas de la série de Bonvarlet [12]. De même un corps étranger intra articulaire a été suspecté chez un de nos patients ayant une SVN localisée largement nécrosée, Bonvarlet et al [12] ont souligné un cas similaire expliquant que les fragments de la tumeur nécrosés se comporteraient comme un corps étranger.

La gêne fonctionnelle cause une amyotrophie quadricipitale rapportée chez 21 des 23 patients dans la série de Flandry [5]. Certaines formes ont des manifestations cliniques trompeuses, la plus déroutante est celle d'un kyste poplité volumineux isolé et chronique. Il peut s'agir comme il a été rapporté [1] d'une extension postérieure de la maladie synoviale diffuse. Néanmoins, l'association d'un kyste poplité et d'une pathologie rhumatismale authentique est classique [1]. Les observations rapportées par Rosenberg [1] en 2001 et par Meehan et Daftari en 1994 [13] dans lesquelles la synovite villonodulaire fut découverte à l'occasion d'un kyste poplité mais dans lesquelles la prolifération villonodulaire intéressait également l'articulation du genou, est tout à fait analogue à celle de notre patient qui avait le kyste synovial et une hydarthrose associée. D'une autre part notre deuxième patient présentait un kyste poplité isolé ce qui peut s'agir d'une localisation postérieure de la prolifération révélant la maladie [1]. La SVN dans sa forme pseudo septique a été observée dans 3 cas, elle est liée à une nécrose de la tumeur par torsion de son pédicule comme en témoigne l'examen anatomopathologique réalisé qui a mentionné cet aspect nécrotique, cette forme a été également individualisée dans 3 cas dans la série de Bonvarlet et al [12]. L'antécédent de traumatisme du genou affecté peut être trouvé dans à peu près 50% des patients [4,5], Sharma [7] trouvait ce facteur dans 25% de ses patients. La radiographie est le plus souvent normale d'autant plus qu'il s'agit d'une forme localisée. Conformément à la littérature on a trouvé 35% des lésions appartenant toutes à des formes diffuses, selon Sharma [7] les lésions osseuses peuvent se voir dans 33% à 56% des formes diffuses [7] Rao [2] en retrouve 67%. L'IRM réalise une cartographie des lésions, le signe le plus caractéristique est la présence d'un signal hétérogène au sein d'un épaississement synovial avec présence d'hypo signal en T1 ou T2 correspondant aux dépôts d'hémosidérine. Elle est sensible mais peu spécifique [1, 6, 7, 10,12], rares sont les faux négatifs [12] quoique 2 cas aient été rencontrés dans notre série. L'histologie reste l'examen de certitude, la SVNP est caractérisée par la prolifération du stroma fibreux, l'infiltration par les histiocytes, et les cellules géantes multi nucléés avec dépôts d'hémosidérine au sein des sidérophages de la synoviale [3, 7,10].

Un cas rencontré dans notre série suscite un intérêt particulier, il s'agit d'une SVN diffuse et proliférative vue à L'IRM, et confirmée par l'histologie, et qui, après 5 mois de la synovectomie subtotale s'est compliquée par l'apparition de gonalgies et de nodules sous cutanés dont l'examen anatomopathologique parlait de synovialosarcome monophasique grade III envahissant l'os mais heureusement sans métastases pulmonaires. S'agit-il d'une dégénérescence maligne ou d'une erreur diagnostique? En fait, Enzinger et Weiss ont défini une forme maligne de synovite villonodulaire pigmentée comme étant soit une prolifération dans laquelle on identifie des lésions de synovite villonodulaire pigmentée et des caractéristiques malignes, soit une prolifération maligne se développant dans un site où a été diagnostiquée auparavant une synovite villonodulaire pigmentée [14]. En se référant à la définition d'Enzinger et Weiss, Bertoni et al [15] ont décrit huit cas qu'ils considéraient comme des synovites villonodulaires pigmentées de type malin En tenant compte de l'évolution fatale de la moitié des patients, ils ont préconisé une chirurgie radicale. Pour distinguer cette entité Bertoni et al [15] ont proposé de se baser sur les critères de malignité suivants : infiltration de la membrane synoviale des tissus mous sous jacents, présence de cellules rondes ou ovales mononuclées avec cytoplasme éosinophiles, noyaux plus larges avec mitoses typiques et atypiques, moins de cellules inflammatoires moins de cellules avec inclusion lipidique, et des zones de nécrose sans cellules inflammatoires. En revanche, le sarcome synovial monophasique est de diagnostic très difficile et nécessite un examen attentif, car il pose souvent un piège diagnostique avec la SVN [3], rendant nécessaire le recours à l'immunohistochimie voire l'étude cytogénétique qui est d'un apport diagnostique réel.10 cas de SVN ont été pris comme des synovialosarcome dans la série de U.Nilsonne et Moberger (1969) [3] qui ont conclue aux mêmes critères de malignité décrites par Bertoni et al [15] pour différencier le synovialosarcome de la SVN. Un cas similaire a été rapporté par D.Gouders (2001) [14] quoique son cas ait fait 6 récidives diagnostiquées à chaque fois comme des SVN à l'examen anatomopathologique, et le diagnostic de sarcome synovial n'a été identifié qu'après la survenue de métastases pulmonaires et péritonéales. En effet l'essentiel de la littérature concerne des cas sporadiques ou de petites séries de cas. La centralisation des données cliniques, histologiques et thérapeutique décrites pour chaque cas rencontré, pourrait probablement nous aider à résoudre cette confusion. Le traitement est fondé sur la synovectomie totale sous peine de récidive pour les formes diffuses, elle sera si possible arthroscopique ou après arthrotomie, ces deux techniques peuvent d'ailleurs être complémentaires pour assurer une exérèse totale. La forme localisée guérit après ablation du nodule [1, 7, 8, 10,12] en cas de lésion diffuse plus invasive la chirurgie doit être étendue, pouvant conduire exceptionnement selon certains auteurs à l'amputation [14]. Les récidives sont une réelle caractéristique de la maladie, plus fréquentes dans les formes diffuses [7, 10,12], Flandry [5] en rapportait 8%, augmentant de 35% après 25 ans.

Sharma et al [7] ont détecté 3 récidives sur 14 formes diffuses, par contre ils n'ont noté aucune récidive pour les formes localisées traitées par l'excision locale, l'efficacité de ce traitement effectué par arthrotomie ou par arthroscopie a été confirmée par l'étude de J.Dines [10] qui s'est basée sur un long suivie de 26 ans et n'a noté aucune récidive. Notre fréquence de récidive est comparable aux résultats publiés. (10% dans les formes diffuses 0% dans les formes localisées).Olgivie et Harris [8] ont conclu que la synovectomie totale abaisse significativement le taux de récidive par rapport à la synovectomie partielle. Dans notre série les récidives sont sous formes diffuses, elles peuvent être localisées, Bonvarlet et al [12] ont noté la récidive de 3 formes localisées sous une forme diffuse, Le Tiec [6] rapportait une récidive sous forme mixte d'une forme diffuse, ce qui montre la transition entre les formes ou l'insuffisance du diagnostic initial et /ou du traitement. Le traitement complémentaire par l'acide osmique, quant il est possible, est diversement apprécié. La durée minimale de la surveillance conseillée est de 5 ans, les récidives surviennent souvent des les 3 premières années [7, 10,14] comme dans un de nos cas. Le pronostic est plus péjoratif en cas d'atteinte ostéocartilagineuse.

## Conclusion

La SVN demeure une pathologie mystérieuse. Son polymorphisme clinique et surtout son diagnostic différentiel avec le synovialosarcome, ainsi que les formes malignes récemment individualisées rendent les options thérapeutiques encore difficiles. D'où l'intérêt d'une bonne coopération avec les radiologues, les anatomopathologistes, les rhumatologues et les kinésithérapeutes pour une meilleure prise en charge.

### Etat des connaissances actuelles sur le sujet

- La synovite villonodulaire est une tumeur synoviale bénigne, rare, d'étiopathogénie inconnue;
- Elle est caractérisée par une symptomatologie clinique non spécifique;
- Cette tumeur à fort pouvoir prolifératif et destructeur pose le problème de diagnostic différentiel particulièrement avec le synovialosarcome.

### Contribution de notre étude à la connaissance

- Confirmation des données épidémiologiques, cliniques et radiologiques de la synovite villonodulaire;
- Possibilité de dégénérescence maligne de la synovite villonodulaire.

## Conflits d’intérêts

Les auteurs ne déclarent aucun conflit d'intérêts.
